# Phenolic profile, alpha-amylase inhibitory activity, and *in vitro* glycemic index of adzuki beans

**DOI:** 10.3389/fnut.2022.1063602

**Published:** 2022-12-22

**Authors:** Mazhar Muhammad, Yong Zhu, Anyan Wen, Na Liu, Likang Qin

**Affiliations:** ^1^Key Laboratory of Plant Resource Conservation and Germplasm Innovation in Mountainous Region (Ministry of Education), College of Life Sciences/Institute of Agro-Bioengineering, Guizhou University, Guiyang, Guizhou, China; ^2^School of Liquor and Food Engineering, Guizhou University, Guiyang, Guizhou, China

**Keywords:** adzuki beans, phenolic compounds, alpha-amylase inhibitory activity, resistant starch, *in vitro* glycemic index

## Abstract

Regular consumption of low-glycemic index (GI) foods is a common strategy for type 2 diabetes patients. To evaluate the potential application of adzuki beans in low-GI foods, the phenolic profile and alpha-amylase inhibitor (α-AI) activity of four varieties of adzuki beans (G24, Te Xiao Li No. 1, Gui Nong No. 1, and Qian Xiao Hei) were determined. The starch digestibility properties and *in vitro* glycemic index (IVGI) of these beans were also evaluated using the *in vitro* digestion model coupled with 3,5-dinitrosalicylic acid colorimetry. The results indicated that these adzuki beans, containing numerous phenolics, showed inhibitory activities to alpha-amylase with the α-AI activities between 1.760 ± 0.044 and 3.411 ± 0.186 U/g. The resistant starch (RS) contributed predominantly to the total starch with proportions between (69.78 ± 2.45%) and (81.03 ± 0.06%); Te Xiao Li No. 1 was the highest compared with the other varieties. The adzuki beans were categorized into low- or medium-GI foods, and the IVGI ranged from (39.00 ± 0.36) to (56.76 ± 4.21). These results suggested that adzuki beans can be used as a component of low-GI foods.

## Graphical Abstract

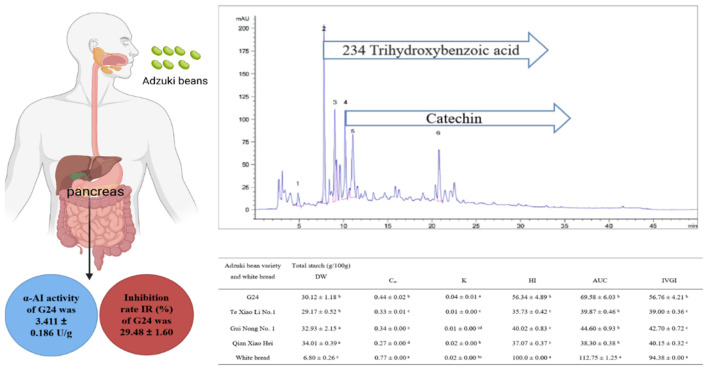

## 1 Introduction

Adzuki beans (*Vigna angularis*) are common legumes appearing in red, white, black, and green colors (1, 2). China is the largest cultivator and distributes these beans to Japan, Korea, the Philippines, and Malaysia (3). Owing to their delicious taste, these beans are widely used as ingredients in desserts and served at various celebrations, and processed adzuki beans are used more excessively compared with their raw counterpart (4). Adzuki beans contain an abundance of resistant starch (RS), which provides health benefits to type 2 diabetes patients (5, 6), and the RS contributes approximately 50% to the total starch (TS) in beans (7). RS presents resistance to gut digestive enzymes, and is fermented by anaerobic intestinal microbiota in the colon, producing short-chain fatty acids, which offer beneficial effects on type 2 diabetes (8). In addition, adzuki beans are an abundant source of phenolics, and gallic acid, *p*-hydroxybenzoic acid, ferulic acid, and catechin have been identified in beans (9, 10). These may inhibit the activities of α-amylase and α-glucosidase, frustrating the degradation of polysaccharides into oligosaccharides and monosaccharides (11–13). The α-AI is another dominant component which also inhibits the activities of alpha-amylase (14). Previous studies showed that beans containing functional components presented a lower glycemic index (GI), of which the bean containing total phenolics (61.50 ± 2.12 g/100 g) presented a GI of 55.23 (15), and the bean with RS proportion at 55.60% presented the GI at 60.0 (16), another study indicated that kidney beans classifying in low GI item contained abundant phenolics and RS, which also presented inhibitory activities to alpha-amylase with the specific activity range at 1.65 ± 0.05–4.16 ± 0.04 U/g (17).

Type 2 diabetes is one of the common chronic diseases, which is caused by a disorder of the human pancreas (β-cells). In normal human pancreatic β-cells, pancreatic insulin is stimulated by incretins, such as glucagon-like peptide-1 (GLP-1) and glucose-dependent insulinotropic peptide (GIP), which are responsible for glucose homeostasis (18). Previous studies have shown that adzuki beans are low-GI foods, which positively affects insulin sensitivity and resistance (19–21). In addition, an epidemiological study revealed that regular consumption of common beans can reduce the risk of type 2 diabetes (22). GI is defined as the response of blood sugar after consumption of foods that contain a definite quantity of carbohydrates compared with a reference food (white bread or glucose) (23, 24). Diets with elevated GIs are associated with a high risk of cancer (25), type 2 diabetes (26), and heart disease (27, 28).

Adzuki bean is a staple crop in Bijie city of Guizhou province, where the typical geography conditions are hilly topographic and changeable climate which induce the production of endogenous phytochemicals (29), and the adzuki beans were adopted as food supplements for hyperglycemic patients in the local area, the excellent varieties with a higher yield were selected for the functional evaluation, in which the phenolic profiles were determined using high-performance liquid chromatography (HPLC), the starch digestibility and inhibitory activities to the α-amylase were also determined, the potential GI was evaluated using *in vitro* digestion model, and the contribution of endogenous components to low-GI was also discussed. All of these results provide the fundamentals for the mechanism revealing that adzuki beans regulate the blood glucose content.

## 2 Materials and methods

### 2.1 Sample preparation

**FIGURE 1:**
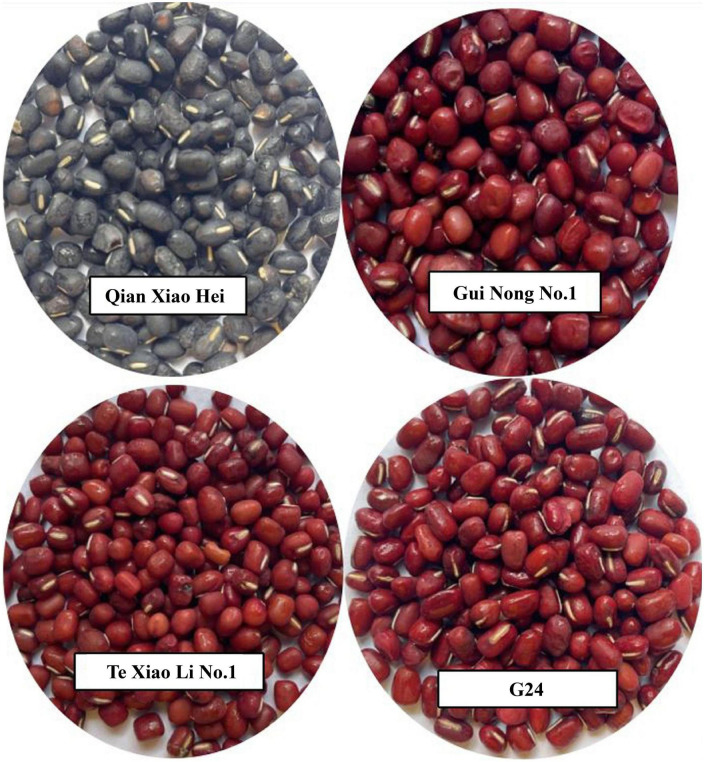
Four varieties of adzuki beans (G24, Te Xiao Li No. 1, Gui Nong No. 1 and Qian Xiao Hei).

The four varieties of adzuki beans, namely G24, Te Xiao Li No. 1, Gui Nong No. 1, and Qian Xiao Hei (Figure 1), planted at the breeding base (27.32°N, 105.29°E, 1,640 m altitude), were supplied by He You Xun, Bijie Institute of Agricultural Science, Guizhou Province, China. In the growth cycle, the average temperature was 19^°^C, the precipitation was approximately 780.6 mm, and the sunshine duration was 667 h. The adzuki beans were ground into flour using a universal high-speed smashing machine (Dongguan Xinmeihua Electromechanical Equipment Co., Ltd., Dongguan City, P. R. China), and then screened using a 40-mesh sieve; the flours were stored at –20°C for further analysis.

### 2.2 Chemicals

Folin-Ciocalteu’s phenol reagent, pepsin (source: gastric mucosa of a pig), and gallic acid were purchased from Sigma-Aldrich Ltd. (St. Louis, MO, USA). 3, 5-Dinitrosalicylic acid (DNS) was purchased from Sinopharm Chemical Reagent Co., Ltd. Acetone and hydrochloric acid (36–38%) were purchased from Chongqing Chuandong Chemical Group Co., Ltd. (Chongqing, P. R. China). Sodium hydroxide was purchased from Chengdu Jinshan Chemical Reagent Co., Ltd. (Chengdu, P. R. China). Deionized water was purchased from Watsons Water Co., Ltd. (Guangzhou, P. R. China). HPLC grade acetonitrile and acetate were purchased from Aladdin company (Shanghai, P. R. China). Sodium carbonate was purchased from Yongda Chemical Reagent Co., Ltd. (Tianjin, P. R. China). Aluminum chloride hexahydrate was purchased from Kemio Chemical Co., Ltd. (Tianjin, P. R. China). Rutin, 2, 3, 4-trihydroxybenzoic acid, catechin, chlorogenic acid, *p*-hydroxybenzoic acid, and ferulic acid were purchased from Macklin Bioch. Co., Ltd (Shanghai, P. R. China). Alpha-amylase enzyme (type: pancreatic, source: *Bacillus*) was purchased from Solarbio Science & Tech. Co., Ltd. (Beijing, P. R. China). Hexane, ethyl acetate, acetic acid, petroleum ether, and methanol were purchased from Tianjin Fuyu Fine Chemical Co., Ltd. (Tianjin, P. R. China).

### 2.3 Extraction of phenolics

Phenolic extracts were obtained using the method described by Adom and Liu (30) and De La Parra et al. (31). In brief, 4 g of whole adzuki beans powder was homogenized in 15 ml of chilled acetone (80%, v/v) for 15 min. These mixtures were centrifuged (CenLee16R, Hunan Cenlee Scientific Instrument Co., Ltd., Hunan, China) at 8,000 × *g* for 5 min to collect the supernatants. The residues were again subjected to phenolic extraction twice following the procedures mentioned above, and the collected supernatants were evaporated at 45°C using a rotary evaporator (Shanghai Yarong Biochemical Instrument Factory) and eventually reconstituted to 10 ml with deionized water. The phenolic extracts were stored at –20^°^C for further analysis, and the phenolic extraction was performed in triplicate.

### 2.4 Determination of total phenolics

Total phenolics (TP) were determined using the method described by De La Parra et al. (31). A volume of 100 μl of gallic acid standard and adzuki bean extracts were mixed with 400 μl deionized water and 100 μl of Folin-Ciocalteu phenol reagent. The mixtures were kept at room temperature for 6 min, and subsequently mixed with 1 ml of 7% Na_2_CO_3_ solution and 0.8 ml of deionized water. After 90 min of reaction in the dark, 200 μl of the reaction mixtures were pipetted into a 96-well microplate reader (SPECTRAMAX 190, Molecular Devices, Silicon Valley, USA) and determined at 760 nm, and the standard curves were plotted from the absorbances and concentrations of the gallic acid standards. The TP was calculated from the standard curve and expressed as mg gallic acid equivalents (GAE)/100 g DW.

### 2.5 Determination of total flavonoids

The total flavonoids (TF) were estimated using the methods described by Zhishen et al. (32) and Bakar et al. (33). Five hundred microliters of adzuki bean extracts or rutin standard was allowed to react with 2.25 ml of deionized water and 0.15 ml of 5% NaNO_2_ for 6 min; the mixtures were continuously reacted with 0.3 ml of 10% AlCl_3_.6H_2_O for 5 min. Afterward, 1 ml of 1 mol/L NaOH was vigorously mixed with the mixtures to terminate the reactions. After 15 min of reaction, 200 μl of the mixtures was pipetted into a 96-well microplate reader and determined at 510 nm. The standard curve was plotted from the absorbance and concentration of the rutin standards, and the TF was calculated from the standard curve and expressed as mg rutin equivalents (RE)/100 g DW.

### 2.6 Profile of phenolic compounds

The profile of the phenolic compounds of the adzuki beans was determined using HPLC (34, 35). The adzuki bean extracts were directly passed through a 0.45-μm filter film, and then the filtered extracts (20 μl) were injected into a 1260 HPLC device (1260 Infinity, Agilent Technologies, Santa Clara, CA, USA) equipped with a C-18 chromatography column (30°C) (Agilent ZORBBAX 250 mm × 4.6 mm, 5 μm). Acetonitrile (A) and 1% acetic acid (B) solutions were obtained using the gradient elution program: 0–5 min, 5–15% A; 5–35 min, 15–35% A; 35–40 min, 35–45% A; 40–50 min, 45–5% A, with a flow rate of 1 ml/min. These individual phenolic compounds were detected using an ultraviolet absorption detector at 280 nm. The individual phenolic compounds were identified and quantified according to the retention time and calibration curve of the corresponding standards. The individual phenolic compound contents were expressed as mg phenolics/100 g DW.

### 2.7 Determination of α-AI activity

Four grams of adzuki beans powder was adequately mixed with 20 ml of deionized water at 25°C for 1 h. Next, the mixtures were centrifuged (8,000 × *g*, 4°C) for 30 min to collect the water extracts, and then the volume was recomposed to 20 ml. The above process was performed in triplicate (23).

The α-AI specific activity was determined using the method described by Yang et al. (14), with minor modifications. First, the water extracts were appropriately diluted to keep the α-amylase inhibition rate (IR) under 50%. Second, 0.25 ml of diluted water extracts were adequately mixed with 0.25 ml of α-amylase solution (1 U/ml) and 0.5 ml of PBS (pH 6.9) in a water bath (37^°^C) for 10 min, and then a 5-min reaction was allowed after the addition of 0.5 ml of 1% (w/v) aqueous starch solution to the mixtures. Eventually, the mixtures were mixed with 1 ml DNS solution and subsequently heated in a boiling water bath for 10 min. After cooling to room temperature, the volume of the mixtures was reconstituted to 20 ml with deionized water and measured at 540 nm using a spectrophotometer instrument (L5S, Shanghai Yidian Analytical Instrument Co. Ltd., Shanghai, China).

The trial groups were estimated as (1) blank tube (no addition of water extracts), (2) blank control tube (addition of neither α-amylase solution nor water extracts), and (3) inhibition control tube (no addition of α-amylase solution). PBS was used to make up for the deficiency.

The inhibition rate of the α-AI in adzuki beans to α-amylase was calculated using formula (1):


(1) IR%=[1-(A-3A)4/(A-1A)2]×100%

Where *A_1_, A_2_, A_3_*, and *A*_4_ are the absorbances of the blank tube, blank control tube, inhibition tube, and inhibitory control tube at 540 nm wavelength, respectively. The amount of α-amylase that catalyzes the formation of 1 mol glucose/min at 37°C and pH 6.9 was defined as an activity unit (U). The amount of α-AI that catalyzes the inhibition of 1 mol glucose formation per min in an α-amylase catalytic starch hydrolysis reaction at 37°C and pH 6.9 was defined as an activity unit (U), while the α-AI specific activity (U/g) is defined as the α-AI activity unit in 1 g of adzuki bean, and was calculated using formula (2):


(2) α⁢-⁢A⁢I⁢(U/g)=(I⁢R×1×n×V)/m

Where “*1*” represents the activity of α-amylase solution (U/ml), “*n*” is the water extract dilution times, “*V*” is the total volume of water extract (ml), and “*m*” is the mass of the adzuki beans powder (g).

### 2.8 Determination of total starch

The TS of adzuki beans was estimated by referring to the National Food Safety Standards of China GB 5009.9 (36). Adzuki bean powder was first processed in fat to remove sugar, and then it underwent continuous processes, such as gelatinization, saccharification, acid hydrolysis, and neutralization, to obtain the mixtures adopted for TS determination using the alkaline copper tartrate solution titration method.

### 2.9 *In vitro* digestion and glucose content determination

In brief, an aliquot of bean powder comprising 50 mg of starch was mixed with 2 ml of deionized water, and then the mixture was heated for 10 min in a boiling water bath. In addition, 10 ml of HCl-KCl buffer (0.1 mol/L, pH 1.5) and 0.2 ml pepsin solution (1 mg/ml, dissolved in 0.1 mol/L of HCl-KCl buffer) were sequentially added to the gelatinized mixture, which was then agitated for 60 min in a water bath at 40°C. Furthermore, 15 ml of PBS (pH 6.9) and 5 ml of α-amylase solution (2.6 U/ml) (pH 6.9) were sequentially added to the mixture, which was then agitated for 180 min in a water bath at 37°C, and aliquots of the digested mixture (1 ml) were collected at intervals of 30 min (0, 30, 60, 90, 120, 150, and 180 min) for GI determination. The white bread was subjected to the above processes except for the gelatinization process (37). The other aliquots of the digested mixture (1 ml) were collected for 0, 20, and 120 min to determine the digestive properties of the starch (38). The adzuki beans were processed in triplicate. These mixtures were instantly heated to denature the α-amylase activity; eventually, the collected digestion mixtures were sequentially cooled and centrifuged (4°C, 8,000 × *g*, 10 min) before glucose content determination. The DNS colorimetry method was adopted to determine glucose content. The aliquots of the digestion mixture were equivalently mixed with DNS (200 μl). In addition, the mixtures were sequentially processed by heating in boiled water for 6 min and cooling in running water to room temperature before recomposing the volume to 2 ml using deionized water. The absorbances of the reacted mixtures were measured using a spectrophotometer at 540 nm.

### 2.10 Calculation of starch digestibility properties

The slowly digestible starch (SDS), rapidly digestible starch (RDS), and RS contents were calculated using formula (3), formula (4), and formula (5) (38, 39).


(3) RDS(%)=[(C-20C)0×V×0.9/50]×100

(4) SDS(%)=[(C-120C)20×V×0.9/50]×100

(5) RS(%)=1-[(RDS+SDS)/50]×100

Where *C*_0_ represents the initial glucose concentration (mg/ml) before the enzymatic hydrolysis reaction, “*C*_20_” and “*C*_120_” represent the glucose concentration (mg/ml) released within 20 and 120 min, respectively, “*V*” refers to the initial volume of the enzymatic hydrolysis, “*0.9*” is the conversion factor of glucose to starch, and “*50*” represents the starch content (mg) in the measured sample.

### 2.11 Calculation of the *in vitro* glycemic index

The IVGI of adzuki beans was computed as described in previous studies (23, 37). The starch hydrolysis curves were plotted, and the enzymatic hydrolysis time and starch hydrolysis rate were designated as the abscissa and ordinate, respectively.

The starch hydrolysis rate (SR%) was calculated using formula (6):


(6) SR(%)=[(C-tC)0×V×0.9]/50×100%

“*C*_*t*_” represents the glucose concentration at a time “*t*” (mg/ml), “*C*_0_” represents the glucose concentration at a time “*0*” (mg/ml), “*32.2*” represents the initial volume of the enzymatic hydrolysis reaction system (ml), “*0.*9” is the conversion factor of glucose to starch, and “*50*” represents the amount of starch in adzuki bean powder.

Formula (7) was used to calculate the enzymatic hydrolysis kinetic constant (k) and the infinite glucose content (C_∞_) (mg/ml).


(7) C-tC=0C(1-exp(-kt))∞

The area under the hydrolysis curve (AUC) was delineated using formula (8).


AUC=C(t-ft)0∞-(CK∞/)[1-exp(-k(t-ft)0)]

Where “*t*_*f*_” and “*t*_0_” represent the last time point (180 min) and the initial time point (0 min) of the enzymatic hydrolysis reaction, respectively.

The IVGI was computed using formula (9).


(9) GI=(0.862×calcHI×100)+8.198

The calcHI-starch hydrolysis index indicates the AUC sample/AUC white bread standard.

### 2.12 Statistical analysis

The values were expressed as means ± standard deviation (*n* = 3) and tested using the IBM SPSS (one-way ANOVA plus *post-hoc* Tukey’s b test) software (IBM, NY, USA). *P*-values less than 0.05 indicated a significant difference.

## 3 Results

### 3.1 Total phenolics, total flavonoids, and phenolic profile

**FIGURE 2:**
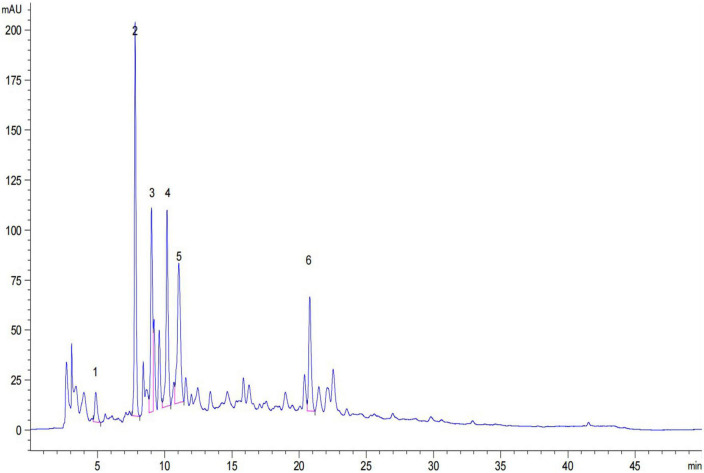
High-performance liquid chromatography (HPLC) chromatogram of individual phenolic compounds in adzuki beans. 1, gallic acid; 2, 2, 3, 4-trihydroxybenzoic acid; 3, chlorogenic acid; 4, catechin; 5, rutin and 6, *p*-hydroxybenzoic acid.

The TP and TF of adzuki beans are shown in Table 1. The TP ranges from (137.11 ± 4.81) to (154.90 ± 4.45) mg GAE/100 g DW; Te Xiao Li No. 1 has the highest TP content, followed by Qian Xiao Hei, G24, and Gui Nong No. 1. The TF ranges from (279.78 ± 2.54) to (602.00 ± 10.23) mg RE/100 g DW; G24 has the highest TF content. In addition, gallic acid, 2, 3, 4-trihydroxybenzoic acid, chlorogenic acid, catechin, rutin, and *p*-hydroxybenzoic acid were identified in adzuki beans, as shown in Table 2 and Figure 2. The 2, 3, 4-trihydroxybenzoic acid, chlorogenic acid, and *p*-hydroxybenzoic acid contents range from (1.19 ± 0.13) to (7.46 ± 0.75) mg/100 g DW, (3.94 ± 0.27) to (7.15 ± 1.08) mg/100 g DW, and (5.96 ± 0.59) to (7.54 ± 0.16) mg/100 g DW, respectively. The catechin and rutin contents range from (12.63 ± 1.38) to (23.05 ± 1.60) mg/100 g DW and (2.41 ± 0.19) to (16.31 ± 2.09) mg/100 g DW, respectively. In Te Xiao Li No. 1, the rutin and catechin contents are dominant with 16.31 ± 2.09 and 23.05 ± 1.60 mg/100 g DW, respectively.

**TABLE 1:** Total phenolics (mg GAE/100 g DW), total flavonoids (mg RE/100 g DW), and moisture contents (%) of adzuki beans.

Sample	MC	TP	TF
< /*ltx*:*thead* > *G*24	13.55 ± 0.07^a^	144.79 ± 5.07^ab^	602.00 ± 10.23^a^
*TeXiaoLiNo*.1	13.87 ± 0.12^a^	154.90 ± 4.45^a^	549.93 ± 14.00^b^
*GuiNongNo*.1	13.94 ± 0.18^a^	137.11 ± 4.81^b^	279.78 ± 2.54^d^
*QianXiaoHei*	14.12 ± 0.72^a^	145.53 ± 3.77^ab^	353.51 ± 8.95^c^

MC, moisture content; TP, total phenolics; TF, total flavonoids. The values were expressed as means ± SD, *n* = 3, some of which in the same column with no same letters indicate significant differences at the level of *p* < 0.05.

**TABLE 2:** Phenolic compound profiles (mg/100 g DW) of adzuki beans.

Sample	GA	2,3,4-THBA	CA	R	*p*-HBA	C
G24	ND	1.19 ± 0.13^b^	3.94 ± 0.27^b^	5.74 ± 0.30^b^	5.96 ± 0.59^b^	12.63 ± 1.38^b^
Te Xiao Li No. 1	0.71 ± 0.03^a^	7.46 ± 0.75^a^	7.15 ± 1.08^a^	16.31 ± 2.09^a^	ND	23.05 ± 1.60^a^
Gui Nong No. 1	ND	4.46 ± 0.24^b^	ND	2.41 ± 0.19^c^	7.54 ± 0.16^a^	ND
Qian Xiao Hei	0.73 ± 0.04^a^	ND	6.72 ± 0.81^a^	ND	6.14 ± 0.15^b^	ND

GA, gallic acid; 2,3,4-THBA, 2, 3, 4-trihydroxybenzoic acid; CA, chlorogenic acid; R, rutin; *p*-HBA, *p*-hydroxybenzoic acid; C, catechin; ND, not detected. The values were expressed as means ± SD, *n* = 3, some of which in the same column with no same letters indicate significant differences at the level of *p* < 0.05.

### 3.2 α-AI specific activity

**FIGURE 3:**
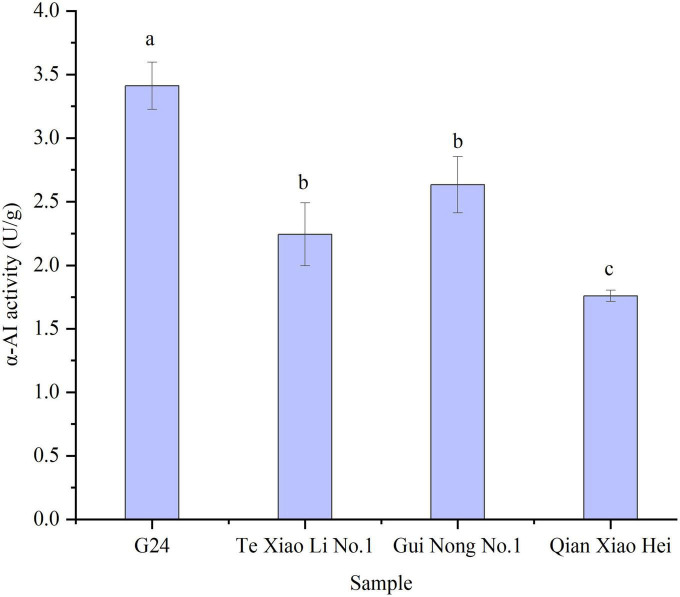
The α-AI specific activities of adzuki beans. Bars with no same letters indicate significant differences at the level of *p* < 0.05.

The α-AI specific activity indicates the inhibitory efficiency of α-AI to α-amylase in the starch hydrolysis reaction. The α-AI specific activities of adzuki beans range from (1.76 ± 0.04) to (3.41 ± 0.18) U/g. G24 shows the highest activity and Te Xiao Li No. 1 and Gui Nong No. 1 show minor fluctuations; both are ranked intermediate, while Qian Xiao Hei shows the lowest α-AI specific activity (Figure 3).

### 3.3 Starch digestibility properties

The starch digestibility properties can be categorized using the digestive process in which the RDS can be digested in 0–20 min, the SDS can be digested in 20–120 min, and the RS cannot be digested after 120 min (39). In adzuki beans, the proportions of RS to TS range from (69.78 ± 2.45%) to (81.03 ± 0.06%); Te Xiao Li No. 1 has the highest proportion of RS to TS, followed by Gui Nong No. 1, Qian Xiao Hei, and G24. The proportions of SDS to TS are between (11.02 ± 1.58%) and (15.57 ± 0.17%), and the proportions of RDS to TS are between (3.38 ± 0.12%) and (18.62 ± 1.81%) (Table 3).

**TABLE 3:** The *in vitro* starch digestibility of adzuki beans.

Sample	RDS (%)	SDS (%)	RS (%)
G24	18.62 ± 1.81^a^	15.34 ± 0.71^a^	69.78 ± 2.45^d^
Te Xiao Li No. 1	3.38 ± 0.12^b^	15.57 ± 0.17^a^	81.03 ± 0.06^a^
Gui Nong No. 1	10.95 ± 1.82^a^	11.02 ± 1.58^b^	78.02 ± 0.52^b^
Qian Xiao Hei	9.46 ± 1.14^a^	15.34 ± 0.25^a^	75.19 ± 1.25^c^

RDS, rapidly digestible starch; SDS, slowly digestible starch; RS, resistant starch. The values were expressed as means ± SD, *n* = 3, some of which in the same column with no same letters indicate significant differences at the level of *p* < 0.05.

### 3.4 *In vitro* glycemic index

**FIGURE 4:**
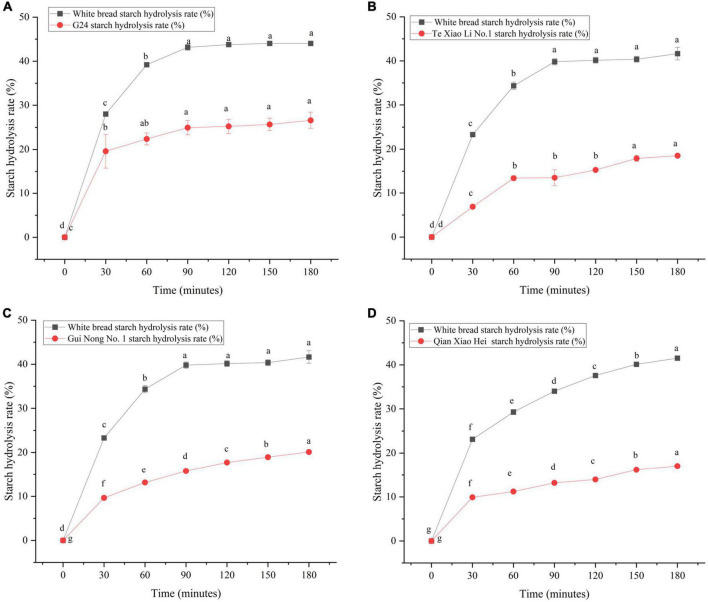
Dynamic changes of starch hydrolysis rate of adzuki beans. Panel **(A)** exhibits the starch hydrolysis curves of G24 and white bread, panel **(B)** exhibits the starch hydrolysis curves of Te Xiao Li No. 1 and white bread, panel **(C)** exhibits the starch hydrolysis curves of Gui Nong No. 1 and white bread, and panel **(D)** exhibits the starch hydrolysis curves of Qian Xiao Hei and white bread. Bars in the same curves with no same letters indicate significant differences at the level of *p* < 0.05.

The SR was determined using the *in vitro* digestion model coupled with DNS colorimetry, which was used to plot the starch hydrolysis curves (Figures 4A–D). Using the curves, the enzymatic hydrolysis kinetic constant (k) and the infinite glucose content (C_∞_) were obtained, and both were eventually used to calculate the GI; the parameters and IVGI are presented in Table 4. The SR of adzuki beans at 180 min ranges from (17.02 ± 0.33%) to (26.60 ± 1.81%). Qian Xiao Hei, Gui Nong No. 1, and Te Xiao Li No. 1 present a low SR in the whole hydrolysis period. The IVGI of adzuki beans ranges from (39.00 ± 0.36) to (56.76 ± 4.21), and Te Xiao Li No. 1 presents the lowest IVGI.

**TABLE 4:** *In vitro* glycemic index and involved parameters of adzuki beans.

Sample	TS (g/100 g) DW	C_∞_	K	AUC	HI	IVGI
G24	30.12 ± 1.18^b^	0.44 ± 0.02^b^	0.04 ± 0.01^a^	69.58 ± 6.03^b^	56.34 ± 4.89^b^	56.76 ± 4.21^b^
Te Xiao Li No. 1	29.17 ± 0.52^b^	0.33 ± 0.01^c^	0.01 ± 0.00^c^	39.87 ± 0.46^b^	35.73 ± 0.42^c^	39.00 ± 0.36^c^
Gui Nong No. 1	32.93 ± 2.15^a^	0.34 ± 0.00^c^	0.01 ± 0.00^cd^	44.60 ± 0.93^b^	40.02 ± 0.83^c^	42.70 ± 0.72^c^
Qian Xiao Hei	34.01 ± 0.39^a^	0.27 ± 0.00^d^	0.02 ± 0.00^b^	38.30 ± 0.38^b^	37.07 ± 0.37^c^	40.15 ± 0.32^c^
White bread	6.80 ± 0.26^c^	0.77 ± 0.00^a^	0.02 ± 0.00^bc^	112.75 ± 1.25^a^	100.0 ± 0.00^a^	94.38 ± 0.00^a^

TS, total starch; C_∞_, infinite glucose content; K, enzymatic hydrolysis kinetic constant; AUC, area under the hydrolysis curve; HI, starch hydrolysis index; IVGI, *in vitro* glycemic index. The values were expressed as means ± SD, *n* = 3, some of which in the same column with no same letters indicate significant differences at the level of *p* < 0.05.

## 4 Discussion

Abundant phenolics were identified in the seed coats of beans, and intrinsic anthocyanins exist in colored beans (40, 41). Phenolic acids and flavonoids are the dominant subclasses of phenolic compounds; phenolic acids can be divided into two major categories, namely hydroxybenzoic acids and hydroxycinnamic acids (42). Flavonoids are polyphenolic-secondary metabolites containing a 15-carbon atom structure in which phenyl rings (A and B) are coupled with a heterocyclic ring (C) (43). Phenolic acids and flavonoids are the dominant phenolics in plant-based foods, and flavonoids existing in free and glycosidic forms are the most common type in plants (40). In the present study, the TP of adzuki beans was higher than that of red and black beans in a previous study (44). In the present study, the TP of adzuki beans was lower than their counterpart reported by previous studies (45, 46), while the TP of common beans (0.72–1.04 mg GAE/g) reported by a previous study (47) was lower than that reported by the present study. The TP and TF of various adzuki beans ranged from (2.11 ± 0.01) to (2.75 ± 0.04) GAE/g of DW and (59.17 ± 2.06) to (70.41 ± 1.32) RE/g of DW, respectively (48), which were higher than those of the present study. In addition, the TF of G24 was higher than that of green beans (49). Previous studies have suggested that the consumption of beans can provide numerous health benefits to humans, such as lowering the risks of cardiovascular disease, obesity, type 2 diabetes, and different types of malignant growths (50, 51). Thus, beans also possesses inhibitory activities to α-amylase and α-glucosidase (52, 53), which are positively associated with type 2 diabetes.

The 2, 3, 4-trihydroxybenzoic acid and chlorogenic acid contents of Te Xiao Li No. 1 were higher than those of legumes as reported by a previous study, in which gallic acid was the dominant phenolic with the contents between (0.39 ± 0.00) and (479.26 ± 10.40) μg/g (45), whereas the gallic acid content of Te Xiao Li No. 1 and Qian Xiao Hei was 0.71 ± 0.03 and 0.73 ± 0.04 mg/100 g DW, respectively. Moreover, gallic acid was not detected in a previous study (46). The variations may be attributed to the differences in varieties, genomics, geographical, and environmental conditions (54, 55). Previous studies have suggested that gallic acid, chlorogenic acid, *p*-hydroxybenzoic acid, and caffeic acid in beans have positive influences on type 2 diabetes (56, 57). In the present study, Te Xiao Li No. 1 presented the highest catechin and rutin contents; a previous study suggested that catechin in dietary beans presented an inhibitory response toward fungal amylase (58). Furthermore, catechin showed inhibitory activities to α-amylase, lipase, and α-glucosidase, which is directly linked with blood sugar content (10, 59, 60).

Alpha-amylase is the key enzyme that hydrolyzes carbohydrates to disaccharides and oligosaccharides in the gastrointestinal tract, both of which are further degraded into monosaccharides or simple sugars by α-glucosidase, resulting in an increased level of postprandial glucose (61). α-AI (α-AI-1, α-AI-2, α-AI-3, and α-AI-L) is a functional component of beans, which presents anti-hyperglycemic activities. The most common isoforms of α-AIs isolated from beans are α-AI-1, α-AI-2, and α-AI-L (62–66). α-AI is a glycoprotein containing active inhibitory subunits, which are named α- and β-chains; the α-chain lacks tryptophan and the β-chain lacks methionine, while cysteine is absent in both of them (67, 68). Previous studies have suggested that phenolics also show inhibitory activity to α-amylase (15, 69), which indicates that the α-AI specific activity of adzuki beans may be attributed to glycoprotein and phenolics. A previous study indicated that the water extract of adzuki beans presents an α-AI activity to α-amylases and α-glucosidases, providing health benefits to type 2 diabetes patients (70). Alpha-amylase contains an active site for digesting starch, which is completely blocked by α-AI (62); the quantity of the inhibitor that colonizes the active site should be defined. A certain amount of sample is required to maintain the inhibition rate of enzymes (71).

Alpha-D-glucopyranosyl linked with α-D- (1–4) or α-D- (1–6) linkages are the basic units of starches, which are composed of amylose and amylopectin (72, 73). Previous studies have shown that the abundant RS in legumes is associated with postprandial blood glucose content, contributing to a positive effect on type 2 diabetes (74, 75). In the present study, the proportions of RS to TS in adzuki beans ranged from (69.78 ± 2.45%) to (81.03 ± 0.06%), which was higher than the results reported by previous studies (38, 76, 77). RS can hardly be digested in the stomach and small intestine (8), and it is eventually fermented by gut microbiota (78), producing short-chain fatty acids in the colon, which impose positive effects on type 2 diabetes (21, 79).

GI indicates the sugar level in the blood after the consumption of certain foods, and can be classified into three categories, namely low GI (<55), medium GI (56–69), and high GI (70–100) (80). In the present study, adzuki beans can be classified as low- or medium-GI foods and the IVGI ranges from (39.00 ± 0.36) to (56.76 ± 4.21); Te Xiao Li No. 1 presented the lowest IVGI, and the IVGI of adzuki beans in ascending order is Qian Xiao Hei < Gui Nong No. 1 < G24. The GIs of these adzuki beans were lower than those of whole beans (81), mung beans (82), and adzuki beans (83). Previous studies have suggested that phenolics impose positive effects on postprandial blood glucose levels in aspects of inhibiting amylase activities, decreasing glucose absorption, and stimulating insulin secretion. Furthermore, phenolics may also reduce the glucose content by suppressing glucose release in the liver, which is regulated by the intracellular signal pathway (84). The IVGIs of these adzuki beans may be due to the whole effects of endogenous functional components. Phenolics, α-amylase inhibitors, and RS are the dominant contributors to the delays in blood sugar responses after the consumption of adzuki beans. In addition, the IVGI of Qian Xiao Hei (black-colored) was comparable with Te Xiao Li No. 1 (red-colored); however, the phenolic profile, α-AI specific activity, and RS proportion of Qian Xiao Hei were not close to those of Te Xiao Li No. 1. This finding indicates that Qian Xiao Hei may contain some unidentified endogenous components, which may contribute to low GI.

## 5 Conclusion

In the present study, adzuki beans contain numerous phenolics, including gallic acid, 2, 3, 4-trihydroxybenzoic acid, chlorogenic acid, catechin, rutin, and *p*-hydroxybenzoic acid; of which catechin is the dominant phenolic compound in Te Xiao Li No. 1 and G24 varieties. The TP of Te Xiao Li No. 1 is higher than that of the other varieties, and the TF of G24 is higher than that of the other varieties. In addition, α-amylase inhibitors and RS are also present in adzuki beans. These adzuki beans are classified as low- or medium-GI foods, which may be caused by the whole effects of endogenous functional components. Phenolics, α-amylase inhibitors, and RS are the dominant contributors to the delays in starch digestion. These results indicate that these adzuki beans can be used as components of low-GI foods.

## Data availability statement

The original contributions presented in this study are included in the article/supplementary material, further inquiries can be directed to the corresponding author.

## Author contributions

MM: data curation, validation, writing—original draft, software, formal analysis, and investigation. LQ: investigation, resources, methodology, supervision, conceptualization, project administration, and funding acquisition. YZ: resources, writing—review and editing, and funding acquisition. AW and NL: formal analysis. All authors contributed to the article and approved the submitted version.
